# Kinin Peptides Enhance Inflammatory and Oxidative Responses Promoting Apoptosis in a Parkinson's Disease Cellular Model

**DOI:** 10.1155/2016/4567343

**Published:** 2016-09-18

**Authors:** Anna Niewiarowska-Sendo, Andrzej Kozik, Ibeth Guevara-Lora

**Affiliations:** Department of Analytical Biochemistry, Faculty of Biochemistry, Biophysics and Biotechnology, Jagiellonian University in Krakow, Krakow, Poland

## Abstract

Kinin peptides ubiquitously occur in nervous tissue and participate in inflammatory processes associated with distinct neurological disorders. These substances have also been demonstrated to promote the oxidative stress. On the other hand, the importance of oxidative stress and inflammation has been emphasized in disorders that involve the neurodegenerative processes such as Parkinson's disease (PD). A growing number of reports have demonstrated the increased expression of kinin receptors in neurodegenerative diseases. In this study, the effect of bradykinin and des-Arg^10^-kallidin, two representative kinin peptides, was analyzed with respect to inflammatory response and induction of oxidative stress in a PD cellular model, obtained after stimulation of differentiated SK-N-SH cells with a neurotoxin, 1-methyl-4-phenylpyridinium. Kinin peptides caused an increased cytokine release and enhanced production of reactive oxygen species and NO by cells. These changes were accompanied by a loss of cell viability and a greater activation of caspases involved in apoptosis progression. Moreover, the neurotoxin and kinin peptides altered the dopamine receptor 2 expression. Kinin receptor expression was also changed by the neurotoxin. These results suggest a mediatory role of kinin peptides in the development of neurodegeneration and may offer new possibilities for its regulation by using specific antagonists of kinin receptors.

## 1. Introduction

Neurodegenerative disorders represent a global health problem, being a major cause of high mortality among elderly populations. One of the most common degenerative diseases of the nervous system is Parkinson's disease (PD). It is related to the death of dopaminergic neurons in the basal ganglia of the brain [1]. Regardless of the etiology of this disease, including genetic or environmental factors, inflammatory processes are associated with the progression of neurodegeneration [2, 3]. In fact, several lines of evidence confirm the interdependence between neuroinflammation and neurodegenerative disorders, including an increased cytokine release and an imbalance of molecules involved in oxidative stress [2–5]. These substances, produced mainly by microglial and dendritic cells, act on astrocytes and neurons, inducing secondary responses that can lead to uncontrolled inflammation in nervous tissues, resulting in cell apoptosis. Moreover, the ability of neuronal cells to release inflammatory mediators and reactive oxygen species (ROS) in response to stimulus has also been reported [5]. Therefore, an indirect self-destruction of neurons cannot be excluded in neurodegenerative disorders. Current studies have shown that kinins, potent proinflammatory peptides, can be produced by cells of the nervous system, even by neuronal cells [6–8], and their presence is correlated with neuroinflammation [9–11]. Differentiated and INF-*γ*-stimulated neuroblastoma cell line IMR-32 was able to produce quickly significant amount of bradykinin and its metabolites, the peptides without arginine residue at the C-terminus that act through a specific kinin receptor type 1 (B1R), enhancing inflammatory responses [6]. In addition, an increasing number of studies indicated the involvement of kinin receptors in neurological disorders including those associated with cell degeneration [11–15]. It was demonstrated that kinins can activate not only nonneuronal cells, such as astrocytes and microglia, but also neurons, causing proinflammatory reactions [16–18]. Recently, interesting investigations on anti-inflammatory therapies in Parkinson's disease were reported [19]. Despite the fact that no direct evidence of the participation of kinin peptides in PD has been presented so far, the examination of the impact of these peptides on degenerative processes seems to be interesting. In the present study, we investigated the influence of bradykinin (BK) and des-Arg^10^-kallidin (DAKD) on apoptosis markers (such as caspase activity) or cell viability in a PD cellular model. Neuron-like cells, obtained from retinoid acid-differentiated human neuroblastoma cell line SK-N-SH, treated with a neurotoxin, 1-methyl-4-phenylpyridinium (MPP+), showed enhanced degenerative response. The treatment with kinin peptides enhanced this effect and was accompanied by increased cytokine release and generation of oxidative molecules.

## 2. Materials and Methods

### 2.1. Chemicals

Rabbit polyclonal antibodies against kinin receptor 1, mouse monoclonal antibodies against kinin receptor 2 (B2R), FITC-conjugated goat polyclonal antibodies against rabbit, and TRITC-conjugated rabbit polyclonal antibodies against mouse were supplied by Abcam (England). BK and DAKD were obtained from Bachem (Switzerland). ELISA kits for detection of cytokines, interleukin-1*β* (IL-1*β*), interleukin-6 (IL-6), and tumor necrosis factor-*α* (TNF-*α*), were purchased from BD Biosciences (USA). 2-Mercaptomethyl-3-guanidinoethylthiopropanoic acid (MGTA) was supplied by Calbiochem (USA) and Fluorescence Mounting Medium from DAKO (Denmark). All primers were obtained from Genomed (Poland). SYBR Green Kit for Real-Time PCR was purchased from Kapa Biosystems (USA). A kit for the determination of caspase 3/7 activity (CaspoGlo3) and M-MLV Reverse Transcriptase kit were from Promega (USA). Rabbit polyclonal antibodies against receptor D2 (D2R) were purchased from Santa Cruz Biotechnology (USA). 2,3-Diaminonaphthalene (DAN), 2,7-dichlorodihydrofluorescein diacetate (DCFH2-DA), icatibant (HOE), des-Arg^10^-icatibant (desHOE), captopril, bacitracin, protease inhibitors, retinoid acid (RA), 1-methyl-4-phenylpyridinium (MPP+), resazurin sodium salt, 4,6-diamidino-2-phenylindole (DAPI), Tri-Reagent, and standard chemicals were supplied by Sigma (USA). Antibiotics, antimycotics, cell culture media, fetal bovine serum (FBS), nonessential amino acids, and sodium pyruvate were purchased from Thermo Scientific (USA).

### 2.2. Cell Culture, Differentiation, and PD Cellular Model

The human neuroblastoma cell line SK-N-SH supplied by ATCC (USA) was cultured in MEM medium supplemented with 1% nonessential amino acids, 1 mM sodium pyruvate, 1 U/mL penicillin, 1 *μ*g/mL streptomycin and 2.5 *μ*g/mL amphotericin B, and 10% FBS in a humidified atmosphere containing 5% CO_2_ at 37°C. SK-N-SH cells were differentiated with 5 *μ*M RA into neuronal-like cells according to previously published method [20]. The PD cellular model was obtained by stimulation of RA-differentiated cells with 2.5 mM MPP+ in 1% FBS MEM medium for 24 hours. Changes in neurite length were observed with an inverted phase contrast microscope (Nikon Eclipse TS100, Japan) equipped with ToupCam Industrial Digital Camera (TP605100A, China) before and after stimulation with neurotoxin.

### 2.3. Cell Viability Measurement

The effect of kinin peptides on cell viability in the cellular PD model was evaluated by using AlamarBlue® assay. After differentiation, cells were incubated with BK and DAKD at different concentrations (1 nM or 1 *μ*M) in 1% FBS cell medium supplemented with kininase inhibitors (10 *μ*M MGTA, 20 *μ*M captopril, and 500 *μ*M bacitracin) for 24 hours. On the next day, cells were treated with 2.5 mM MPP+ in fresh medium containing kinin peptides. After further 24-hour incubation, the cell viability was tested. Briefly, the stimulating medium was discarded and 100 *μ*L of 10% resazurin in phenol red-free medium was added to each well. Then, cells were incubated for 4 hours at 37°C and after that time the fluorescence of supernatants was measured using Synergy H1 microplate reader (BioTek Instruments, USA) at 560 nm excitation and 590 nm emission wavelengths. Simultaneously, samples without MPP+ or kinin treatment were also measured.

### 2.4. Caspase Activity Measurement

Caspase 3/7 activity was assayed with a chemiluminescent method using a luminogenic substrate. RA-differentiated cells (6 × 10^3^) were incubated with BK or DAKD at 1 nM or 1 *μ*M concentration for 24 hours. Next, the samples were stimulated as during the viability test (see above) and after further 24 hours caspase 3/7 activity was measured according to the manufacturer's instructions. Chemiluminescence was measured using Synergy H1 microplate reader (BioTek Instruments, USA). Some samples were first preincubated with antagonists for kinin receptors, icatibant (HOE), and des-Arg^10^-icatibant (desHOE) at 1 *μ*M concentration for 2 hours before treatment with kinins and the antagonists were also added during the further incubation with kinins and MPP+.

### 2.5. Gene Expression Analysis

Total RNA from untreated cells or cells incubated with 5 mM MPP+, 1 *μ*M BK, or 1 *μ*M DAKD for several hours was isolated with Tri-Reagent. Expression of mRNA for B1R, B2R, D2R, IL-1*β*, IL-6, inducible NO synthase (iNOS), and TNF-*α* was analyzed with Real-Time PCR procedure. Expression of elongation factor 2 (EF-2) mRNA was also determined in each sample for subsequent quantitative analysis. cDNA was synthesized with M-MLV Reverse Transcriptase kit according to the manufacturer's instructions. Then, the amplification of cDNA was performed with SYBR Green Kit and specific primers, using the 7500 Fast Real-Time PCR System thermocycler (Life Technologies, USA). The primer pair sequences for the analyzed genes are specified in Table 1. The annealing temperature of Real-Time PCR was 58°C for B1R, B2R, and EF-2, 60°C for D2R and IL-6, and 62°C for IL-1*β*, iNOS, and TNF-*α*. The PCR conditions were 95°C for 10 min, followed by 95°C for 15 s, 15 s at the corresponding annealing temperature, and 72°C for 30 s (40 cycles). The measurement was based on a comparison of analyzed gene expression relative to the housekeeping gene expression (*EF-2*). The relative value of gene expression (*Q*) was calculated with 7500 Fast Real-Time Software by using the equation: *Q* = 2^ΔΔC_T_^, where C_T_ is the threshold cycle.

### 2.6. Measurement of ROS Level

Intracellular level of ROS was determined with a fluorometric assay using 2,7-dichlorodihydrofluorescein diacetate (DCFH2-DA) dye sensible to oxidation [21]. SK-N-SH cells (1.3 × 10^5^) were seeded in a clear bottom opaque microplate, precoated with 1% gelatin. After differentiation, cells were pretreated with kinins as described above and next incubated for 30 minutes with 100 *μ*M DCFH2-DA in medium containing 1% FBS. Then, cells were washed three times and stimulated with 1 *μ*M DAKD or 1 *μ*M BK in the presence of 2.5 mM MPP+ or without neurotoxin. The changes in fluorescence signal were measured every 30 seconds during 20 minutes at 37°C, using microplate Synergy H1 reader at excitation and emission wavelengths of 485 nm and 528 nm, respectively.

### 2.7. Measurements of NO Release

A fluorometric assay [22] was used to analyze the influence of kinin peptides on the production of nitrites (one of the final NO metabolites). Confluent RA-differentiated cells were treated with kinins as described above and next incubated with 1 *μ*M BK and 1 *μ*M DAKD in the presence of or without 2.5 mM MPP+ for 15 and 30 minutes. After this incubation time, nitrite concentration was measured in supernatants. Briefly, 100 *μ*L of supernatant was mixed with 10 *μ*L 0.05 mg/mL DAN in opaque microplate and incubated for 30 minutes in the dark. The reaction was stopped by the addition of 5 *μ*L 2.8 M NaOH and fluorescence was measured in Synergy H1 microplate reader at excitation and emission wavelengths of 365 and 450 nm, respectively. The nitrite concentrations were calculated on the basis of two different standard curves (with and without MPP+).

### 2.8. ELISA Measurements of Cytokine Release

SK-N-SH cells (4 × 10^5^) were seeded in a 12-well plate, differentiated for 3 days with RA, and then stimulated for 6 or 24 hours with 1 *μ*M BK or 1 *μ*M DAKD in the presence of 2.5 mM MPP+ or without neurotoxin. Some samples were first preincubated with antagonists for kinin receptors as described above. All experiments were performed with medium supplemented with protease and kininase inhibitors. The assessment of cytokine release (IL-1*β*, IL-6, and TNF-*α*) into the medium was evaluated with ELISA kits according to the manufacturer's instructions. Absorbance was measured with the use of PowerWave microplate reader (BioTek Instruments, USA) at 450 nm.

### 2.9. Immunofluorescence Analysis of Kinin and Dopaminergic Receptors

To determine cell surface receptors, B1R, B2R, and D2R, an immunofluorescence technique with specific labeled antibodies was used. Cells (5 × 10^4^) were seeded on glass cover slides in a 12-well plate and after RA-differentiation were stimulated with 5 mM MPP+ for 1, 2, and 5 hours. Additional experiments aimed to analyze the effect of kinin peptides on D2R expression were also performed. Differentiated cells were incubated with 1 *μ*M BK or 1 *μ*M DAKD for 30 minutes, 1 hour, and 3 hours. Next, following fixation with cold methanol and 1-hour blocking with 2% FBS, cells were incubated overnight at 4°C with 50-fold diluted primary antibodies: rabbit polyclonal anti-B1R, mouse monoclonal anti-B2R, and rabbit polyclonal anti-D2R. After washing, samples were incubated for one hour with diluted (1 : 250) secondary antibodies : goat FITC-labeled anti-rabbit or rabbit TRITC-labeled anti-mouse. Then, 2 *μ*M DAPI was added for 10 minutes and, after extensive washing, cover slides were mounted on microscope slides using Fluorescent Mounting Medium. Negative control samples were prepared omitting the incubation with primary antibodies. The samples were analyzed with epifluorescence DMIRE2 microscope (Leica, Germany) and images were taken at 40x magnification under oil immersion. The intensity of fluorescence was measured in at least three randomly chosen fields of every picture using ImageJ software. The measured area was selected with “brightness slicing” procedure, with a constant lower threshold value. The values for negative control samples were subtracted from the positively labeled slides. The result for control groups was taken as 100% fluorescence intensity.

### 2.10. Statistical Analysis

All values are presented as means ± standard deviation from at least three experiments; the statistical significance was analyzed by Student's *t*-test.

## 3. Results

### 3.1. Kinin Peptides Induce Apoptotic Processes in PD Cell Model

**Figure 1:**
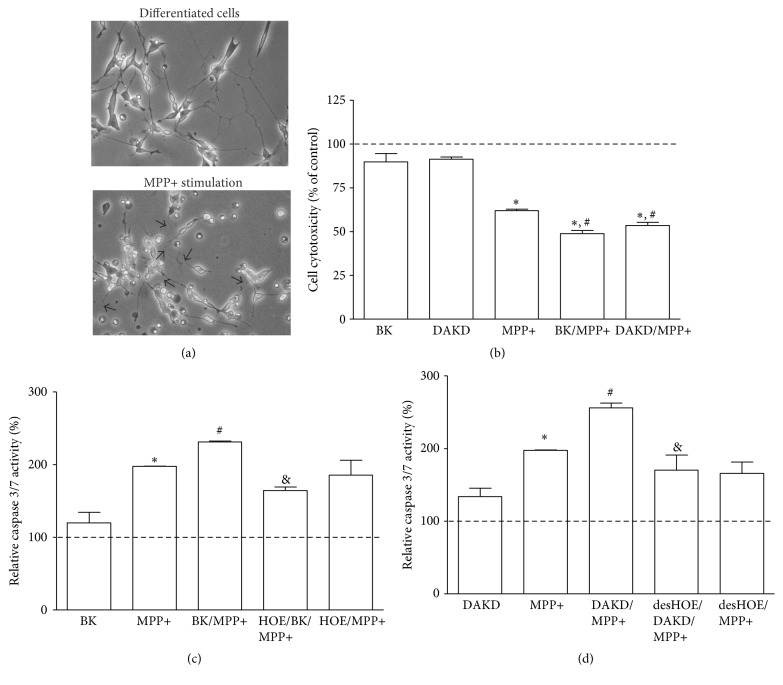
The effect of kinin peptides on apoptotic processes in the PD cellular model. Visualization of the SK-N-SH cells differentiated for 3 days with 5 *μ*M RA with following MPP+ treatment for 24 hours. The destroyed neurites are indicated with arrows (a). RA-differentiated cells after 24 h incubation with 1 *μ*M BK or 1 *μ*M DAKD were stimulated (or not) with 2.5 mM MPP+ for one day. Figures illustrate the percentage cell viability assayed with AlamarBlue test (b) or caspase 3/7 activity analyzed with chemiluminescent assay (c, d) as compared to untreated samples, assumed to show values equal to 100% (dashed line), as described in Materials and Methods. At least three experiments were performed in triplicate. ^*∗*^ *P* < 0.05 versus untreated cells; ^#^ *P* < 0.05 versus MPP+-stimulated cells; ^&^ *P* < 0.05 versus cells treated with kinin peptides and MPP+.

A suitable cellular model of Parkinson's disease was obtained after stimulation of the neuron-like cells with neurotoxin MPP+. The RA-differentiated SK-N-SH cells showed increased neurite degeneration, accompanied by decrease of cell viability and an enhanced activity of caspases after 24-hour incubation with 2.5 mM MPP+ (Figure 1). The well-developed neurite net was broken (see arrows in Figure 1(a)); cells presented changed morphology being more round and some cells were even detached from the plastic support. The cytotoxic effect of neurotoxin on these cells was assayed with AlamarBlue test, showing a significant decrease of cell viability (by 38%), as compared with nonstimulated cells (Figure 1(b)). Kinin peptides, BK as well as DAKD, at the higher concentration (1 *μ*M) caused a slight, insignificant decrease of cell viability (by 10%) while at the lower concentration (1 nM) no toxicity on cells was observed (results not shown). Cells treated with BK and DAKD at 1 *μ*M final concentration were more vulnerable to the MPP+ action. In this case, cell viability was significantly decreased (by ca. 50%) after neurotoxin stimulation. Likewise, meaningful changes in caspase activity were observed after MPP+ stimulation, without and with BK or DAKD treatment (Figures 1(c) and 1(d), resp.). The effect of kinin peptides at 1 nM concentration was smaller (results not shown); thus, in the following experiments only the higher concentration was used. Neuron-like cells, stimulated with neurotoxin, showed an increase in enzyme activity by 100%, as compared to control cells (cells without stimulation were assumed to present 100% of basic enzyme activity). BK and DAKD caused only insignificant increase in caspase activity (by 20% and 32%, resp.). However, these peptides significantly influenced the cell response to MPP+. BK-treated cells showed a 132% augmentation whereas DAKD-treated cells presented an even higher caspase activity after MPP+ coincubation (156%). In addition, the use of antagonists of kinin receptors, HOE and desHOE, produced an inverse effect, suggesting the direct involvement of kinin receptors. Incubation of cells with these substances caused an inhibition of BK- and DAKD-induced caspase activity but it did not affect the MPP+ action significantly.

### 3.2. Effect of Kinin Peptides and MPP+ on Oxidative Stress of Neuron-Like Cells

**Figure 2:**
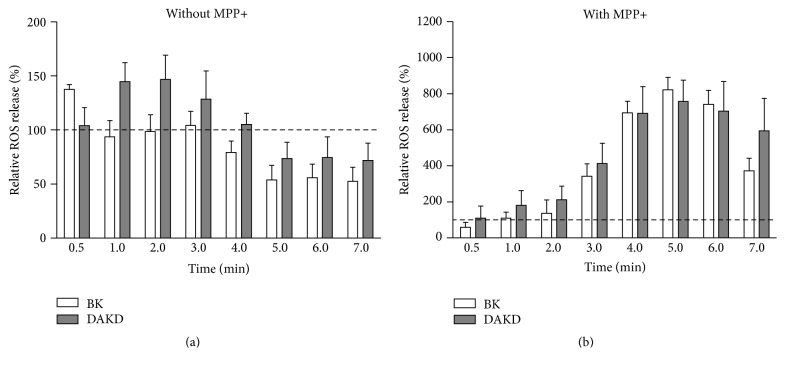
ROS production by differentiated SK-N-SH cells induced by kinin peptides. Cells (1.5 × 10^5^) were previously incubated with kinin peptides for one day. Next, cells were loaded with DCFH2-DA and treated with 1 *μ*M BK or 1 *μ*M DAKD without (a) or in the presence of 2.5 mM MPP+ (b), as described in Materials and Methods. The fluorescence signal was measured for 20 minutes. The figures describe the changes of ROS production as compared with cells untreated with kinin peptides, assumed to show 100% of ROS release (dashed line). At least three experiments were performed in triplicate.

Changes in oxidative processes by kinin peptides were evaluated with fluorometric techniques. The intracellular production of ROS depended on activation time with kinin peptides and on the neurotoxin stimulation. All experiments were performed simultaneously showing time-dependent influence both in neuronal cell model and in PD cellular model. Results are presented as percentage changes induced by peptides compared to values from cells without or with MPP+ stimulation, untreated with kinin peptides (Figures 2(a) and 2(b), resp.). Surprisingly, the response of the cells treated with kinins and MPP+ is much greater (up to eightfold) than that of the cells treated only with kinin peptides. Neuron-like cells showed a quick response, especially to BK. A slight increase of ROS level by BK (by 40%) after 30 seconds was observed while DAKD effect (enhanced ROS production by 50%) appeared later (after 2 minutes). Further incubation showed a tendency of ROS level to decrease even below the basis level detected in control cells. However, after 10-minute incubation the cells achieved a level similar to that observed in untreated cells. In the case of the cellular PD model (cells were additionally incubated with MPP+) the effect of kinin peptides on ROS production was stronger and delayed. The maximal ROS release by both peptides was observed after 5 minutes and increased ca. eightfold as compared with cells treated only with MPP+. The increased ROS level was largely retained especially after DAKD activation, achieving values similar to control cells after 15 minutes from the stimulation start.

**Figure 3:**
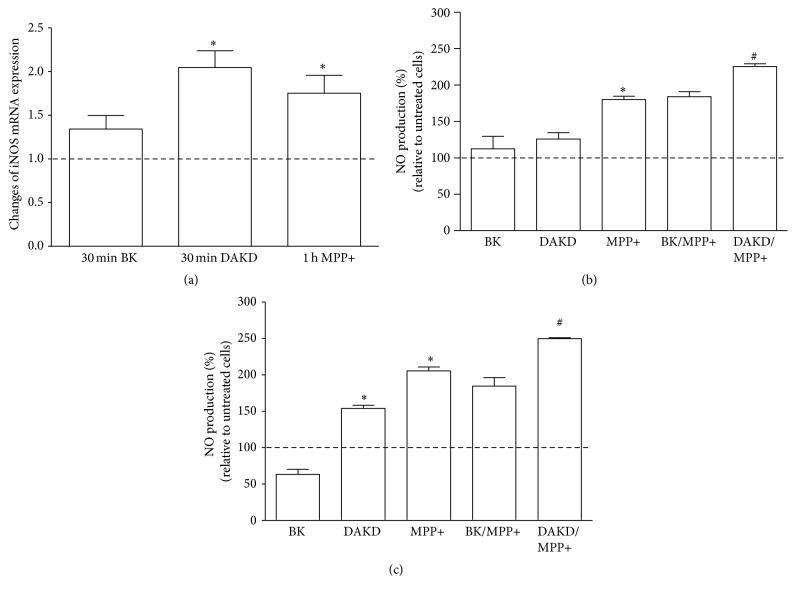
The induction of iNOS mRNA expression and NO production in differentiated SK-N-SH cells by kinin peptides. Confluent cells were treated with 1 *μ*M BK, 1 *μ*M DAKD, or 5 mM MPP+ for different time intervals as described in Materials and Methods and iNOS mRNA expression was analyzed by Real-Time PCR (a). The NO released by confluent cells after treatment with kinin peptides without or in the presence of 2.5 mM MPP+ was analyzed with fluorometric assay, as described in Materials and Methods after 15-minute (b) or 30-minute incubation (c). The bars represent the mean percentage of the changes in comparison to untreated cells, assumed to show values equal to 100% (dashed line). At least three experiments were performed in triplicate. ^*∗*^ *P* < 0.05 versus untreated cells; ^#^ *P* < 0.05 versus MPP+-stimulated cells.

The effect of kinin peptides on oxidative stress in neuron-like cells was also estimated by measuring iNOS mRNA expression and NO release by cells. The iNOS mRNA expression was significantly elevated by DAKD and MPP+ (Figure 3(a)). The DAKD effect was the highest, showing a 2-fold increase of mRNA expression, whereas MPP+ and BK caused an increase in mRNA expression by 75% and 40%, respectively. It should be pointed out that this effect was time-dependent; kinin peptides enhanced mRNA expression of iNOS quickly (after 30 minutes) while the maximum response to neurotoxin appeared after 4-hour incubation. The NO release by neuron-like cells after stimulation with BK, DAKD, and MPP+ is presented in Figures 3(b) and 3(c). No significant effect of BK was detected whereas DAKD caused a significant increase (by 60%) after 30 min incubation. MPP+ induced a higher NO release, by 80% and 100%, after 15-minute and 30-minute incubation, and this effect was significantly enhanced in the presence of DAKD but not of BK. The simultaneous activation with MPP+ and DAKD led to an increase in NO metabolites by 50%, as compared to treatment with MPP+ alone.

### 3.3. Inflammatory Response of PD Cellular Model to Kinin Peptides

**Figure 4:**
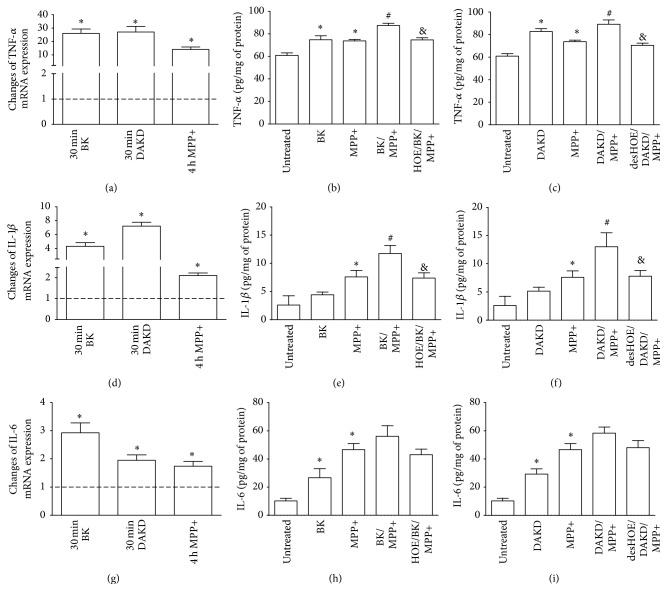
The induced expression of proinflammatory cytokines in differentiated SK-N-SH cells stimulated with kinin peptides. Confluent cell cultures were treated with 1 *μ*M BK or 1 *μ*M DAKD without or in the presence of 2.5 mM MPP+ as described in Materials and Methods. The mRNA expression and protein production for TNF-*α* (a, b, c), IL-1*β* (d, e, f), and IL-6 (g, h, i) were analyzed by Real-Time PCR and ELISA. The figures show the percentage changes of mRNA expression as compared with control samples, assumed to possess 100% expression (dashed line) from at least three experiments performed in duplicate. The amount of released protein was normalized to the amount of sample protein and the results are shown as mean values ± SD from three experiments performed in triplicate. ^*∗*^ *P* < 0.05 versus untreated cells; ^#^ *P* < 0.05 versus MPP+-stimulated cells; ^&^ *P* < 0.05 versus cells stimulated with kinin peptides and MPP+.

The ability of BK and DAKD to induce cytokine expression in neuron-like cells was analyzed by Real-Time PCR and ELISA techniques (Figure 4). Increased mRNA expression for TNF-*α*, IL-1*β*, and IL-6 was registered after just 30 minutes of incubation with peptides. The highest values were obtained for TNF-*α* mRNA expression after 30-minute stimulation with BK and DAKD, showing over 25-fold increase as compared to nonstimulated cells (Figure 4(a)). The effect of MPP+ on the induction of TNF-*α* mRNA in cells was more moderate but significant, showing a 14-fold increase. In addition, the maximal response to neurotoxin was registered after 4-hour incubation. The effect on TNF-*α* protein release by cells after incubation with peptides and MPP+ was quick and after 6 hours protein production was enhanced by 25%, 38%, and 23%, respectively, for BK, DAKD, and MPP+, as compared with untreated cells (Figures 4(b) and 4(c)). Moreover, a slight augmentation of TNF-*α* protein was observed when cells treated with kinin peptides were stimulated with neurotoxin; the amount of this protein was then 20% greater than in cells stimulated with MPP+ alone. In the case of IL-1*β*, kinin peptides (BK, DAKD) and MPP+ caused considerable increase in mRNA expression, 4-, 7-, and 2-fold, respectively (Figure 4(d)). The maximal mRNA expression was observed after 30-minute stimulation with BK and DAKD and after 4-hour neurotoxin incubation. Nevertheless, IL-1*β* protein release after 24 hours was only slightly induced by kinin peptides (1.5-fold and 2-fold for BK and DAKD, resp.) while neurotoxin effect was significantly higher, about 3-fold (Figures 4(e) and 4(f)). Nonetheless, a synergic effect of BK or DAKD with MPP+ on IL-1*β* release was observed. The amount of protein in supernatants was higher than that from cells incubated with neurotoxin alone (by 54% and 72% for BK/MPP+ and DAKD/MPP+, resp.). Besides, the expression of IL-6 mRNA, induced by BK and DAKD, was relatively lower (3-fold and 2-fold, resp.), albeit these changes were significant in comparison to nonstimulated cells (Figure 4(g)). MPP+ caused a slight but significant enhancement increment of IL-6 mRNA (by 1.7-fold). The protein release was also significantly induced by kinin peptides (by 160% and 190% for BK and DAKD treatment, resp.) and by MPP+ (over 3.5-fold) (Figures 4(h) and 4(i)). Similar as in the case of other cytokines, simultaneous cell treatment with kinin peptides and MPP+ also led to an improved protein production (about 420% as compared with untreated cells). The samples treated with antagonists inhibited the production of cytokine proteins, demonstrating that the increment achieved by BK and DAKD in the presence of MPP+ was caused by the activation of the kinin receptors.

### 3.4. The Effect of Neurotoxin MPP+ on the Expression of Dopamine Receptor 2 and Kinin Receptors

**Figure 5:**
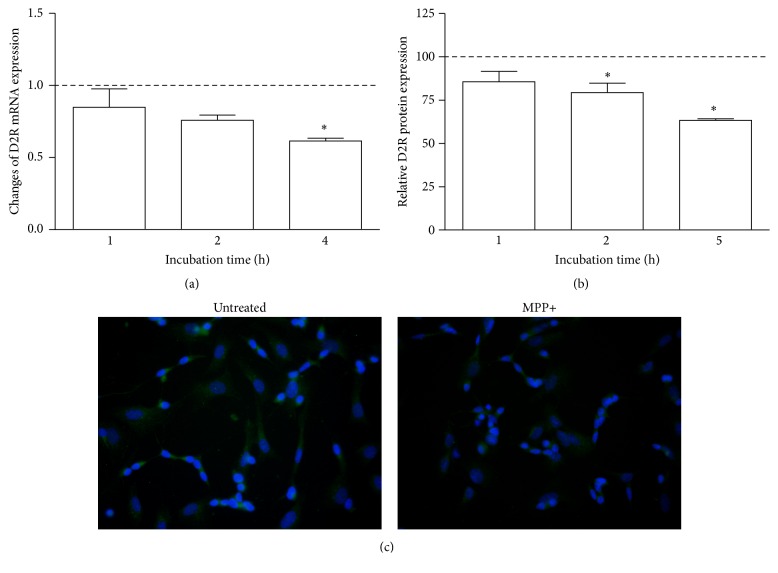
The effect of MPP+ on D2R expression in differentiated SK-N-SH cells. Confluent cells were incubated with MPP+ at different time intervals. D2R mRNA expression was analyzed by Real-Time PCR (a). The figure represents changes of mRNA expression after different times of incubation in comparison to untreated cells assumed to possess value equal to 1 (dashed line). The expression of D2R protein was analyzed by immunocytochemistry with specific antibodies and the quantification of the fluorescence signal was performed as described in Materials and Methods (b). The results are shown as mean ± SD of the changes in comparison to untreated cells, assumed to show 100% expression (dashed line). (c) The photographs show D2R expression by differentiated SK-N-SH cells without or after 5-hour incubation with MPP+. ^*∗*^ *P* < 0.05 versus untreated cells.

**Figure 6:**
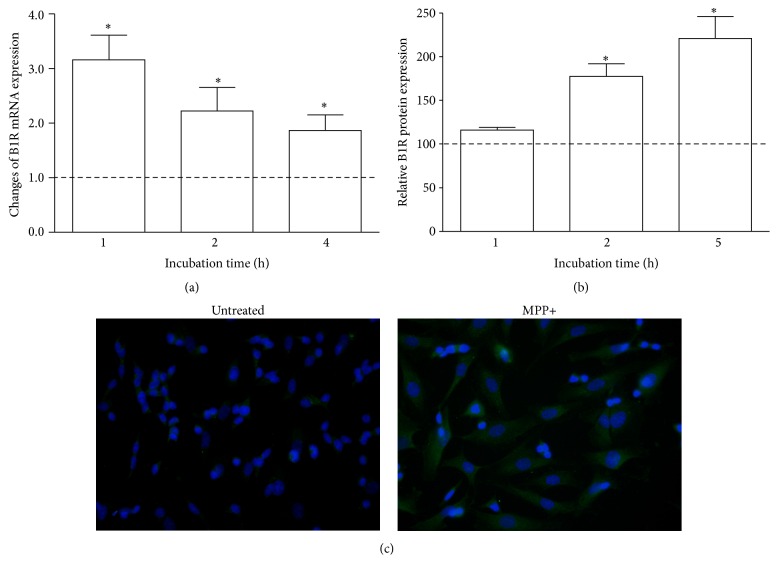
The effect of MPP+ on B1R expression in differentiated SK-N-SH cells. Confluent cells were incubated with MPP+ at different time intervals. (a) The changes of B1R mRNA expression after incubation at different time intervals were analyzed by Real-Time PCR and are presented as relative mean values in comparison to untreated cells, assumed to possess value equal to 1 (dashed line). The expression of B1R protein was analyzed by immunocytochemistry with specific antibodies and the quantification of fluorescence signal was performed as described in Materials and Methods (b). The results are shown as mean ± SD of the changes in comparison to untreated cells, assumed to possess 100% expression (dashed line). (c) The photographs show B1R expression on differentiated SK-N-SH cells without or after 5-hour incubation with MPP+. ^*∗*^ *P* < 0.05 versus untreated cells.

**Figure 7:**
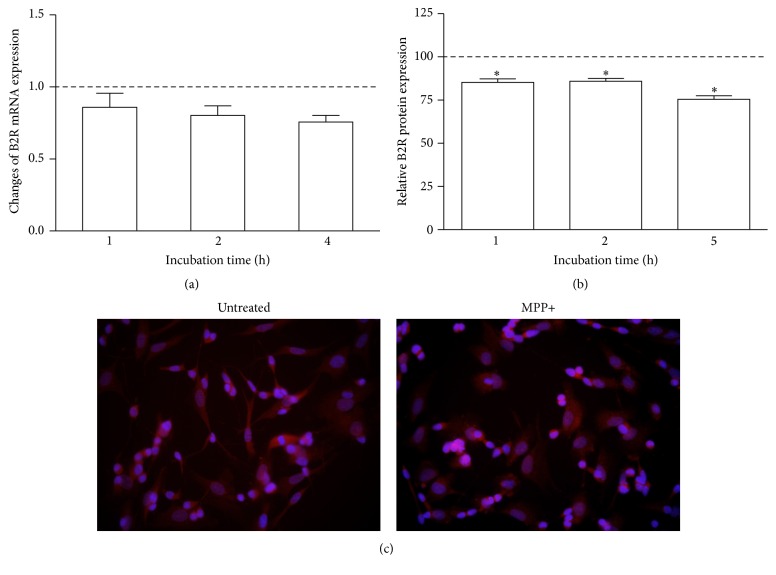
The effect of MPP+ on B2R expression in differentiated SK-N-SH cells. Confluent cells were incubated with MPP+ at different time intervals. (a) Real-Time PCR analysis of the B2R mRNA expression after different incubation time. mRNA expression is shown as relative mean values as compared to untreated cells, assumed to possess value equal to 1 (dashed line). The expression of B2R protein was analyzed by immunocytochemistry with specific antibodies and the quantification of fluorescence signal was performed as described in Materials and Methods (b). The results are shown as mean ± SD of the changes in comparison to untreated cells, assumed to possess 100% expression (dashed line). (c) The photographs show B2R expression by differentiated SK-N-SH cells without or after 5-hour incubation with MPP+. ^*∗*^ *P* < 0.05 versus untreated cells.

The effect of MPP+ on receptors' expression in the neuron-like cells was analyzed at gene and protein level. The D2R mRNA expression by MPP+ was unequivocally decreased (Figure 5). Significant changes were observed after 4-hour incubation when the amount of D2R mRNA decreased by 40% (Figure 5(a)). These observations were in accord with protein expression of this receptor that was diminished to 37% after 5 hours (Figures 5(b) and 5(c)). On the other hand, the expression of kinin receptors was also affected by neurotoxin. The induction of mRNA expression of B1R by MPP+ is undisputable (Figure 6(a)). A significant increase in mRNA expression as compared with control cells (by 200%) was observed after one hour. These changes were preserved after further 3 hours, at a lower but still significant level. Likewise, the amount of B1R protein in the cell membrane was enhanced, leading to above 2-fold augmentation after five hours (Figures 6(b) and 6(c)). Conversely, the expression of B2 receptor in neuronal cells showed a decreasing tendency after neurotoxin treatment (Figure 7). A slight diminution of B2R mRNA was observed after 4 hours (by 20%) but it was not significant as compared to control cells (Figure 7(a)). Nevertheless, the decrease of B2R protein in neuron-like cells observed after MPP+ stimulation (achieving a maximal decrease after 5 hours by 25%) was significant in comparison to untreated cells (Figures 7(b) and 7(c)).

### 3.5. The Effect of Kinin Peptides on the Expression of Dopamine Receptor 2

**Figure 8:**
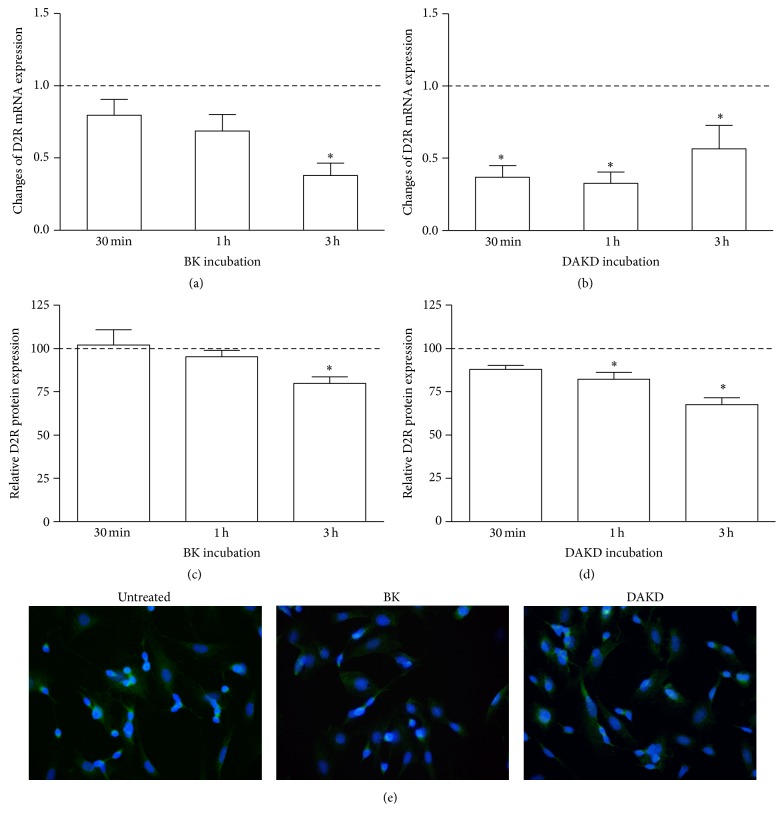
The effect of kinin peptides on D2R expression in differentiated SK-N-SH cells. The mRNA expression of D2R was analyzed with Real-Time PCR after BK (a) or DAKD (b) treatment of confluent cells at different time intervals. D2R mRNA expression is shown as relative mean values as compared to untreated cells, assumed to possess value equal to 1 (dashed line). The expression of D2R protein was analyzed by immunocytochemistry with specific antibodies and the quantification of fluorescence signal was performed as described in Materials and Methods (c, d). The results are shown as mean ± SD of the changes in comparison to untreated cells, assumed to possess 100% expression (dashed line). The photographs show D2R expression on differentiated SK-N-SH cells without or after 3-hour incubation with BK or DAKD (e). ^*∗*^ *P* < 0.05 versus untreated cells.

D2R expression at mRNA and protein level was analyzed after stimulation with kinin peptides at different time intervals. Mainly, cell incubation with BK and DAKD caused a decrease of D2R mRNA (Figure 8). However, this effect was decidedly stronger and significant in the case of DAKD stimulation (Figure 8(b)). BK induced a decrease of mRNA (over 60%) but only after 3-hour incubation (Figure 8(a)), whereas DAKD achieved a similar effect faster, just after 30 minutes of incubation. At the protein level, the cell responses to kinin peptides were comparable (Figures 8(c)–8(e)). The quantitative analysis of the pictures obtained with immunofluorescent technique showed a significant diminution of D2R expression in cells. Changes of the amount of D2R protein were observed after 3-hour BK stimulation (by 20%) and after 1- and 3-hour DAKD stimulation (by 20% and 32%, resp.).

## 4. Discussion

A possibility that tissues of the central nervous system, through the production of inflammation mediators, may contribute to the regulation of neurodegenerative processes [23] incites the reconsideration of these processes in terms of well-known cellular processes associated with inflammation. Kinins and the components necessary for their production are abundantly present in nervous tissues [11, 12, 24]. These substances have been associated with the potentiation of inflammatory responses in nervous tissues [25]. Thus, it is likely that these peptides may contribute to neuronal degeneration by enhancing inflammatory response. Hence, in this study we analyzed the influence of the main kinin peptides, BK and DAKD, in the promulgation of neurodegenerative processes in a cellular model of PD. For this purpose, a popular cellular model related to the human neuroblastoma cell line (SK-N-SH) was differentiated into neuron-like cells with RA [20]. The apoptotic state of these cells was achieved after treatment with MPP+ neurotoxin. The use of this substance as a proapoptotic factor in cellular and animal PD models has been frequently reported [26, 27]. The PD model obtained in this study showed significantly diminished cell viability, with the loss of neurites and enhanced caspase activity (Figure 1). With the help of this model the effect of kinin peptides on apoptosis could be appreciated. Neither BK alone nor DAKD alone did influence significantly the viability of neuron-like cells. However, cell death was significantly enhanced when cells were treated with these peptides before MPP+ incubation, suggesting a better predisposition for neurodegeneration in tissues rich in kinin peptides. In addition, kinin peptides were also able to trigger apoptotic processes through activation of caspases in the PD cellular model. Despite a slight insignificant increment in caspase 3/7 activation by BK and DAKD, these peptides contributed to an enhanced MPP+-induced enzyme activity. Caspase 3 and caspase 7, as executioner enzymes, once activated, can accelerate a feedback loop of caspase activation leading to cell death [28]. The results obtained in this study argue in favor of a proapoptotic effect of both peptides, BK and the active metabolite DAKD, in the PD cellular model. However, a distinct role of B2R and B1R has been proposed in neuronal apoptosis induced by organophosphates [29]. The authors demonstrated that B1R can promote cell damage while B2R may exert a protective role during neurotoxin-induced toxicity. A neuroprotective role of BK against NMDA-mediated excitotoxicity was also suggested [30]. Nevertheless, in our study, the BK-induced effect on the increase of caspase activity in MPP+-treated cells was downregulated by the B2R specific antagonist (HOE), showing an inversion of the BK-induced effect (Figure 1(c)). It should be emphasized that the used concentration of bradykinin was very high, usually considered as pathologic [31]. Hence, the observed effects could take place* in vivo* only if kinin peptides were in abundance in the nervous tissues.

It should be noted that in these experiments cells were treated with BK for extended time before neurotoxin stimulation. Pathologic amount of kinin peptides can stimulate ROS production and cytokine synthesis, even in neuronal tissues [24, 31]. In fact, we were able to observe different effects on ROS production induced by BK and DAKD in the PD cellular model in comparison with the effect induced in neuronal cells (Figure 2). In the latter case, ROS production was immediately induced by kinins and this effect was quickly reversed while in the PD model these peptides caused a much stronger and longer effect. In this context, the difference between our findings and those describing the protective role of BK on cell apoptosis could also be attributed to the different cell response following neurotoxin stimulation. A long exposure of nervous tissues on pathological quantities of BK could make these cells more vulnerable to neurotoxins.

The level of NO in the central nervous system is regulated by a relevant enzyme, neuronal NO synthase, whereas the inducible form (iNOS) is not expressed [32]. However, in pathological situations the expression of this enzyme becomes elevated. Indeed, the mediation of oxidative stress, through ROS and reactive nitrogen species such as NO, in apoptosis is related to a mitochondrial impairment [33]. In addition, acute and chronic oxidative stress may be involved in diverse neurodegenerative diseases, including PD [32]. Experimental models of PD show enhanced iNOS expression with increased NO release. Accordingly, our PD model using MPP+ also displayed increased expression of iNOS mRNA with significant NO production (Figure 3). However, our results demonstrate only a slight increase in iNOS mRNA induced by BK in neuron-like cells, without NO production (Figure 3). In addition, BK was unable to induce NO release also in the PD cellular model. Hence, the observed BK-induced decrease in cell viability and increased caspase activity cannot be unequivocally assigned to the deregulation of oxidative stress. Probably, BK can activate different cellular pathways involved in apoptosis, including those associated with inflammatory response.

The study of inflammatory response in our PD cellular model could provide a hypothesis on BK action. There is emerging evidence that inflammatory processes involving microglia and astrocytes can mediate neurodegeneration, including that in Parkinson's disease [2, 3, 23]. Nevertheless, in this study we demonstrated that neuronal cells could also respond to exogenous stimuli with significant production of proinflammatory molecules. Actually, kinin peptides as well as neurotoxin MPP+ induced a high release of TNF-*α*, IL-1*β*, and IL-6 (Figure 4). In addition, simultaneous treatment of cells with MPP+ and peptides led to enhanced cytokine production. BK evokes a high production of TNF-*α*, IL-1*β*, and IL-6, the cytokines of crucial importance for development of inflammatory response in nervous tissues [34, 35]. Therefore, we can suggest that BK-induced cytokines, especially TNF-*α*, produced by neurons may promote inflammatory processes, causing cell death in our PD cellular model. This assumption is in agreement with the observation obtained in a study using transgenic mice that lacked TNF-*α* receptors; the animals were completely protected against neurotoxicity of MPP+ [36].

The other peptide, DAKD, was able to induce enhanced iNOS mRNA expression in neuron-like cells and also caused overproduction of ROS and NO, which could contribute to starting the extrinsic oxidative stress pathway, especially in the presence of exogenous MPP+ (Figure 3). Like BK, this peptide also caused the release of large amounts of cytokines in neuron-like cells, as well as in the PD cellular model (Figure 4). Hence, our observations argue for the mediation of kinin peptides, especially the B1R agonist, in degenerative processes occurring in the PD cellular model through increased oxidative reactivity and prolonged inflammatory processes.

The dopamine receptor 2 (D2R) plays an important role in Parkinson's disease. Increased D2R expression has been reported in untreated PD patients [37]. However, receptor density and binding potential for agonists gradually decrease during disease progression. The existence of two types of D2 receptor has been reported [37, 38]. The long isoform differs from the short type by the presence of 29 additional amino acids in the third intracellular loop. These receptors possess different functions: the longer receptor is associated with postsynaptic effects, where in cooperation with the dopamine receptor type 1 it modulates the dopaminergic responses producing locomotor activation. On the other hand, the shorter isoform is present, mainly in presynapses, where it acts as an autoreceptor, regulating dopamine secretion by dopaminergic neurons [38]. Here, we report the decrease of D2R expression by MPP+ and suggest that the loss of these receptors implies neurodegeneration. In fact, a protective role of D2 autoreceptors against MPP+ has been reported in* Drosophila* primary neuronal cells [39]. It seems that at the early phase of degenerative processes both receptor D2 isoforms are important for neuroprotection. The lack of postsynaptic D2R may result in degeneration of nervous tissues due to disturbances of the dopaminergic pathways. In turn, the loss of D2 autoreceptors can also lead to cell degeneration because the regulation of dopamine production and release is perturbed, leading to their oxidation by monoamine oxidase that may trigger enhanced redox processes in cells [40]. Therefore, the observed lower expression of D2R mRNA and protein induced by MPP+ corroborate these reports, demonstrating that our cellular model can reflect neurodegenerative features described in PD.

To explain the kinin action in the PD cellular model, the expression of their specific receptors was analyzed after MPP+ stimulation. Kinins and their metabolites without the C-terminal arginine residue (des-Arg kinins) are recognized by two types of receptors, B1R and B2R [13]. The first type of receptor preferably binds des-Arg kinin peptides, whereas B2R primarily recognizes BK and kallidin. The B2R is ubiquitous in numerous types of cells and plays a pivotal role in several physiological processes while B1R is mainly induced by certain stimuli during pathological disorders. Both receptor types may also be involved in inflammatory processes. The B2 receptor plays an important role in acute inflammation while B1R is rather associated with chronic inflammation. This study shows a large increase in B1R mRNA expression that is translated into protein (Figure 6). Therefore, enhanced inflammatory processes in neuronal cells caused by MPP+ are also associated with functioning of this receptor. After activation, it can propagate processes leading to cell degeneration. Interestingly, in the case of the effect of MPP+ on B2R expression, the results were less clear. The mRNA expression of this receptor was not changed by MPP+ (Figure 7). However, a slight decrease of B2R protein after neurotoxin stimulation was observed. These results can indicate, as in the case of D2R, that B2 receptors play an essential role in the protection against MPP+. Indeed, the mediation of BK through B2R in the neuroprotection has recently been proposed [30]. Nevertheless, the expression of B2R is less vulnerable to changes; this receptor after agonist binding and internalization may recycle to the cell surface [13]. In addition, BK can be converted to des-Arg metabolites by carboxypeptidases, present in different tissues, including the brain [6, 41]. Moreover, BK was also able to induce B1R expression on cell membranes [42]. Hence, the observed effect of BK on cell viability and caspase activity can partially be associated with their transformation to des-Arg metabolite, which directly triggers inflammatory and redox reactions leading to neurodegeneration.

Finally, the observations concerning the diminishing of D2R expression by kinin peptides (Figure 8) may confirm a hypothesis of the mediation of these peptides in neurodegenerative processes related to PD. The major effects were observed for the action of DAKD, the B1R agonist. These observations, combined with those obtained from the effect of MPP+ on B1R expression, allowed us to attribute a main role in the development of PD to the des-Arg peptides. The effect of BK could be also attributed to des-Arg bradykinin formation, since in these experiments kininase inhibitors were not used. However, the role of BK in neurodegeneration is not absolutely clear at present and this issue requires additional, more profound studies.

## 5. Conclusions

In this study we examined the effect of kinin peptides on inflammatory and oxidative reactions in a PD cellular model. Kinins as well as their metabolites without arginine residue at the C-terminus were able to induce significant changes in the release of ROS and proinflammatory cytokines. These changes are accompanied by intensified apoptotic processes. Induced expression of B1R by MPP+ may also contribute to a potentiation of inflammatory response. This, in turn, may also enhance cell death processes. Greater abundance of kinin peptides in nervous tissues, produced by neurons as well as by astrocytes and microglia, promotes degeneration of nervous tissue during neuroinflammation. The mediation of kinin receptors may be critical for offering new possibilities for the development of effective therapies against neurodegeneration through their agonists/antagonists.

## Figures and Tables

**Table 1:** Sequences of primers used for Real-Time PCR analysis.

	Forward primer	Reverse primer
B1R	GCAACTGAACGTGGCAGAA	GCCCAAGACAAACACCAGATC
B2R	GTGCCCATGCCGCTTGCTCC	TCGGCGCTGAAAGAGGCCGT
D2R	CTCTTCGGACTCAATAACGCA	CTTTAGTGGAGCCCTCAGGT
EF-2	GACATCACCAAGGGTGTGCAG	GCGGTCAGCACACTGGCATA
IL-1*β*	GATGTCTGGTCCATATGAACTG	TTGGGATCTACACTCTCCAGC
IL-6	CCACAAGCGCCTTCGGTCCA	CTGGGGGTACTGGGGCAGGG
iNOS	TGAACTACGTCCTGTCCCCT	CTCTTCTCTTGGGTCTCCGC
TNF-*α*	TCCTTCAGACACCCTCAACC	AGGCCCCAGTTTGAATTCTT

## Abbreviations

B1R: Kinin receptor 1
B2R: Kinin receptor 2
BK: Bradykinin
DAKD: des-Arg^10^-Kallidin
DAN: 2,3-Diaminonaphthalene
DCFH2-DA: 2,7-Dichlorodihydrofluorescein diacetate
desHOE: des-Arg^10^-Icatibant
D2R: Dopamine receptor 2
EF-2: Elongation factor 2
HOE: Icatibant
IL-1*β*: Interleukin-1*β*
IL-6: Interleukin-6
iNOS: Inducible NO synthase
MGTA: 2-Mercaptomethyl-3-guanidinoethylthiopropanoic acid
MPP+: 1-Methyl-4-phenylpyridinium
PD: Parkinson's disease
RA: Retinoic acid
ROS: Reactive oxygen species
TNF-*α*: Tumor necrosis factor-*α*.

## References

[1] Davie C. A. (2008). A review of Parkinson's disease. *British Medical Bulletin*.

[2] Hirsch E. C., Vyas S., Hunot S. (2012). Neuroinflammation in Parkinson's disease. *Parkinsonism and Related Disorders*.

[3] Khandelwal P. J., Herman A. M., Moussa C. E.-H. (2011). Inflammation in the early stages of neurodegenerative pathology. *Journal of Neuroimmunology*.

[4] Emerit J., Edeas M., Bricaire F. (2004). Neurodegenerative diseases and oxidative stress. *Biomedicine & Pharmacotherapy*.

[5] Akiyama H., Barger S., Barnum S., Bradt B. (2000). Inflammation and Alzheimer's disease. *Neurobiology of Aging*.

[6] Guevara-Lora I., Majkucinska M., Barbasz A., Faussner A., Kozik A. (2011). Kinin generation from exogenous kininogens at the surface of retinoic acid-differentiated human neuroblastoma IMR-32 cells after stimulation with interferon-*γ*. *Peptides*.

[7] Bergamaschini L., Parnetti L., Pareyson D., Canziani S., Cugno M., Agostoni A. (1998). Activation of the contact system in cerebrospinal fluid of patients with Alzheimer disease. *Alzheimer Disease and Associated Disorders*.

[8] Fernando L. P., Natesan S., Joseph K., Kaplan A. P. (2003). High molecular weight kininogen and factor XII binding to endothelial cells and astrocytes. *Thrombosis and Haemostasis*.

[9] Calixto J. B., Cabrini D. A., Ferreira J., Campos M. M. (2000). Kinins in pain and inflammation. *Pain*.

[10] Levant A., Levy E., Argaman M., Fleisher-Berkovich S. (2006). Kinins and neuroinflammation: dual effect on prostaglandin synthesis. *European Journal of Pharmacology*.

[11] Guevara-Lora I. (2012). Kinin-mediated inflammation in neurodegenerative disorders. *Neurochemistry International*.

[12] Raidoo D. M., Bhoola K. D. (1998). Pathophysiology of the kallikrein-kinin system in mammalian nervous tissue. *Pharmacology & Therapeutics*.

[13] Leeb-Lundberg L. M. F., Marceau F., Müller-Esterl W., Pettibone D. J., Zuraw B. L. (2005). International union of pharmacology. XLV. Classification of the kinin receptor family: from molecular mechanisms to pathophysiological consequences. *Pharmacological Reviews*.

[14] Viel T. A., Lima Caetano A., Nasello A. G. (2008). Increases of kinin B1 and B2 receptors binding sites after brain infusion of amyloid-beta 1–40 peptide in rats. *Neurobiology of Aging*.

[15] Costa-Neto C. M., Dillenburg-Pilla P., Heinrich T. A. (2008). Participation of kallikrein–kinin system in different pathologies. *International Immunopharmacology*.

[16] Murakami M., Ohta T., Ito S. (2008). Interleukin-1*β* enhances the action of bradykinin in rat myenteric neurons through up-regulation of glial B1 receptor expression. *Neuroscience*.

[17] Noda M., Kariura Y., Amano T. (2003). Expression and function of bradykinin receptors in microglia. *Life Sciences*.

[18] Wang Y. B., Peng C., Liu Y. H. (2007). Low dose of bradykinin selectively increases intracellular calcium in glioma cells. *Journal of the Neurological Sciences*.

[19] Qian L., Flood P. M., Hong J.-S. (2010). Neuroinflammation is a key player in Parkinson's disease and a prime target for therapy. *Journal of Neural Transmission*.

[20] Niewiarowska-Sendo A., Patrzalek K., Kozik A., Guevara-Lora I. (2015). The effect of differentiation agents on inflammatory and oxidative responses of the human neuroblastoma cell line SK-N-SH. *Acta Biochimica Polonica*.

[21] Wang H., Joseph J. A. (1999). Quantifying cellular oxidative stress by dichlorofluorescein assay using microplate reader. *Free Radical Biology & Medicine*.

[22] Misko T. P., Schilling R. J., Salvemini D., Moore W. M., Currie M. G. (1993). A fluorometric assay for the measurement of nitrite in biological samples. *Analytical Biochemistry*.

[23] Lucas S.-M., Rothwell N. J., Gibson R. M. (2006). The role of inflammation in CNS injury and disease. *British Journal of Pharmacology*.

[24] Naffah-Mazzacoratti M. D. G., Furtado Gouveia T. L., Rodrigues Simoes P. S., Perosa S. R. (2014). What have we learned about the kallikrein-kinin and renin-angiotensin systems in neurological disorders?. *World Journal of Biological Chemistry*.

[25] Black P. H. (2002). Stress and the inflammatory response: a review of neurogenic inflammation. *Brain, Behavior, and Immunity*.

[26] Baratchi S., Kanwar R. K., Kanwar J. R. (2011). Survivin mutant protects differentiated dopaminergic SK-N-SH cells against oxidative stress. *PLoS ONE*.

[27] Kim-Han J. S., Antenor-Dorsey J. A., O'Malley K. L. (2011). The parkinsonian mimetic, MPP^+^, specifically impairs mitochondrial transport in dopamine axons. *The Journal of Neuroscience*.

[28] McIlwain D. R., Berger T., Mak T. W. (2013). Caspase functions in cell death and disease. *Cold Spring Harbor perspectives in biology*.

[29] Torres-Rivera W., Pérez D., Park K.-Y. (2013). Kinin-B2 receptor exerted neuroprotection after diisopropylfluorophosphate-induced neuronal damage. *Neuroscience*.

[30] Martins A. H., Alves J. M., Perez D. (2012). Kinin-B2 receptor mediated neuroprotection after NMDA excitotoxicity is reversed in the presence of kinin-B1 receptor agonists. *PLoS ONE*.

[31] Bhoola K. D., Figueroa C. D., Worthy K. (1992). Bioregulation of kinins: kallikreins, kininogens, and kininases. *Pharmacological Reviews*.

[32] Koppula S., Kumar H., Kim I. S., Choi D.-K. (2012). Reactive oxygen species and inhibitors of inflammatory enzymes, NADPH oxidase, and iNOS in experimental models of parkinsons disease. *Mediators of Inflammation*.

[33] Singh S., Dikshit M. (2007). Apoptotic neuronal death in Parkinson's disease: involvement of nitric oxide. *Brain Research Reviews*.

[34] Thornton P., Pinteaux E., Gibson R. M., Allan S. M., Rothwell N. J. (2006). Interleukin-1-induced neurotoxicity is mediated by glia and requires caspase activation and free radical release. *Journal of Neurochemistry*.

[35] De Vries H. E., Kuiper J., De Boer A. G., Van Berkel T. J. C., Breimer D. D. (1997). The blood-brain barrier in neuroinflammatory diseases. *Pharmacological Reviews*.

[36] Sriram K., Matheson J. M., Benkovic S. A., Miller D. B., Luster M. I., O'Callaghan J. P. (2002). Mice deficient in TNF receptors are protected against dopaminergic neurotoxicity: implications for Parkinson's disease. *The FASEB Journal*.

[37] Hisahara S., Shimohama S. (2011). Dopamine receptors and Parkinson's disease. *International Journal of Medicinal Chemistry*.

[38] Usiello A., Baik J.-H., Rougé-Pont F. (2000). Distinct functions of the two isoforms of dopamine D2 receptors. *Nature*.

[39] Wiemerslage L., Schultz B. J., Ganguly A., Lee D. (2013). Selective degeneration of dopaminergic neurons by MPP^+^ and its rescue by D2 autoreceptors in *Drosophila* primary culture. *Journal of Neurochemistry*.

[40] Jones D. C., Gunasekar P. G., Borowitz J. L., Isom G. E. (2000). Dopamine-induced apoptosis is mediated by oxidative stress and is enhanced by cyanide in differentiated PC12 cells. *Journal of Neurochemistry*.

[41] Guevara-Lora I., Blonska B., Faussner A., Kozik A. (2013). Kinin-generating cellular model obtained from human glioblastoma cell line U-373. *Acta Biochimica Polonica*.

[42] Guevara-Lora I., Florkowska M., Kozik A. (2009). Bradykinin-related peptides up-regulate the expression of kinin B1 and B2 receptor genes in human promonocytic cell line U937. *Acta Biochimica Polonica*.

